# Drug‐related: A case of idiopathic mesenteric phlebosclerosis combined with calcification of the esophageal veins

**DOI:** 10.1002/jgh3.13027

**Published:** 2024-01-17

**Authors:** Chang Liu, Li Li

**Affiliations:** ^1^ Department of Gastroenterology People's Hospital of Chongqing Liang Jiang New Area Chongqing China

**Keywords:** drug‐related, esophageal vein calcification, idiopathic mesenteric phlebosclerosis

## Abstract

Idiopathic mesenteric phlebosclerosis (IMP) is characterized by mesenteric vein calcification and ischemic bowel disease. We describe a unique clinical case of IMP in a patient with a history of oral administration of various drugs, including traditional Chinese medicines (mainly selfheal), vitamin D, and calcium supplements. The disease was not diagnosed in its early stages and was later detected because of the initial symptoms of chest tightness and difficulties in swallowing. During medical examination, esophageal venous sinuses were found through gastroscopy, and CT revealed thickening and widespread calcification of the colonic wall (esophageal wall calcification). Moreover, typical purple‐brown changes in the colonic mucosa were found during colonoscopy. Microscopic examination showed more foam phagocyte, focal lymphocyte aggregation, small‐vessel proliferation, and surrounding collagen‐like deposition which is a typical finding of IMP. More specifically, the patient's mesenteric veins and colon veins were calcified, and the calcification extended to the esophageal veins. These findings were related to long‐term use of traditional Chinese medicines (mainly selfheal). It is possible that excessive intake vitamin D and calcium supplementation may have played a role in the occurrence of vascular calcification, which might have exacerbated the progression of IMP disease.

## Graphical abstract

We describe a unique clinical case of IMP in a patient with a history oftraditional Chinese medicines (mainly selfheal), vitamin D, and calcium supplements.The patient's mesenteric veins and colon veins were calcified, and the calcification extended to the esophageal veins. These findings were related to long‐term use of traditional Chinese medicines. It is possible that excessive intake vitamin D and calcium supplementation may have played a role in the occurrence of vascular calcification, which might have exacerbated the progression of IMP disease.

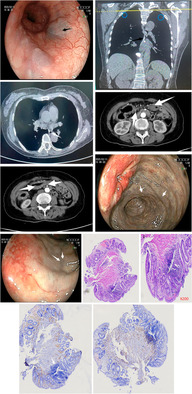

## Introduction

Idiopathic mesenteric phlebosclerosis (IMP) is a rare disease mainly found in East Asia. It is a type of ischaemic colitis caused by arterial sclerosis, thrombosis, or embolism, associated with potentially life‐threatening changes. IMP develops as a result of mesenteric vein calcification with involvement of the colonic mucosal sublayer calcification. The clinical manifestations of IMP include abdominal pain, diarrhea, vomiting, and black, tarry stools. Some patients may be asymptomatic in the early stages of the disease, whereas in the late stage patients may experience intestinal obstruction or perforation. CT scans, colonoscopy, and histopathology are essential tools for an accurate diagnosis of IMP. The treatment of IMP varies from conservative approaches to timely surgical intervention depending on the severity of the disease.^1^

## Cases report

A 68‐year‐old female patient was admitted to the hospital with a 3‐year history of retrosternal obstruction with dysphagia, and her symptoms worsened within a month. She also had acid reflux, nausea, throat discomfort, dry stools, and constipation, but no abdominal pain, bloating, heartburn, diarrhea, vomiting, or bloody stools. She had no history of smoking or drinking. The schistosomiasis was in non‐endemic areas. The patient suffered from hypertension and diabetes, had previously undergone cholecystectomy and tonsillectomy, and was diagnosed with vertebral compression fracture and osteoporosis 2 months prior to hospitalization. She had been intermittently taking a mix of traditional Chinese medicines for the preceding 3 years (mainly selfheal) and had also started taking calcium carbonate and vitamin D (active vitamin D [1,25 hydroxy]) supplements 2 months earlier for osteoporosis. Physical examination revealed that she had upper abdominal tenderness but no rebound tenderness or muscle tension, and no other significant findings.

**Figure 1:**
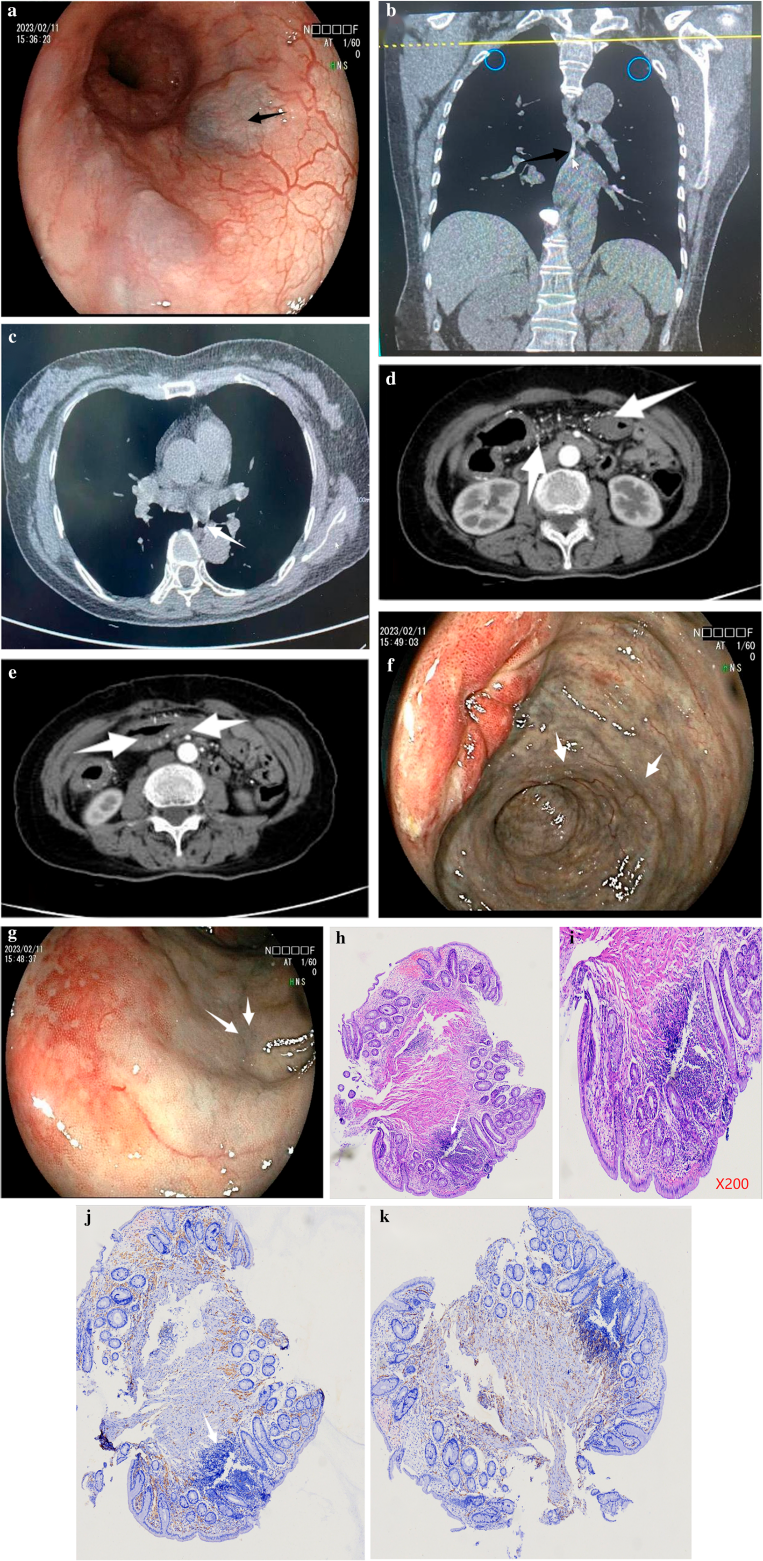
(a) Endoscopy showing multiple esophageal venous sinus (black arrow). (b, c) Computed tomography enterography showing the calcification of the esophageal wall (white arrow). (d, e) Computed tomography enterography showing diffused thickening of the colon with surrounding multiple vascular calcifications (white arrow). (f, g) Endoscopy showing purple mucosa from the cecum to the splenic flexure. The mucosal surface was purplish‐brown with local congestion as observed (white arrow). (h–k) Microscopic images showing (white arrow) inflammatory cell infiltration in the mucosa lamina propria and mucosa intramuscular, more foam phagocyte, focal lymphocyte aggregation, small‐vessel proliferation, and surrounding collagen‐like deposition. (h, i) hematoxylin–eosin staining; (j, k) pathological immunohistochemical staining.

Gastroscopy revealed multiple esophageal venous sinuses (Fig. 1a), which pose a risk of bleeding. Esophageal pressure measurement, 24‐h esophageal pH monitoring, and biopsy were not considered for inspection. Chest CT showed multiple calcification of the esophageal veins (Fig. 1b,c). Enhanced CT of the abdomen revealed diffuse thickening of the caecum, right hemicolon, hepatic flexure of the colon, transverse colon, splenic flexure of the colon, and upper left colon intestinal wall, with multiple visible vascular calcifications around them. Mild enhancement was seen in the intestinal wall on enhanced scans, and no obvious stenosis was seen in the intestinal lumen (Fig. 1d,e). Colonoscopy found tiny ulcers in the rectum, a background of cyanotic mucosa from cecum to sigmoid, dark‐purple edematous mucosa, and dilated veins (Fig. 1f,g). Recumbent radiographs of the abdomen revealed multiple spots and linear high‐density shadows along the colon in the mid‐upper abdomen. Histopathological examination showed inflammatory cell infiltration in the mucosa lamina propria and mucosa intramuscular, more foam phagocyte, focal lymphocyte aggregation, small‐vessel proliferation, and surrounding collagen‐like deposition (Fig. 1h–k).

In light of the typical combination of endoscopic and radiological findings, the final medical diagnosis was determined as IMP. The patient was advised to immediately discontinue the herbal drugs and calcium supplementation. She was diagnosed with chest obstruction and swallowing difficulties as the main symptoms, with no signs of portal hypertension, tumors, autoimmune diseases, tuberculosis, or inflammatory bowel disease. She was put on omeprazole, gastric mucosal protective agents, mesalazine, probiotics, and glutamine. She was also advised to undergo regular colonoscopy follow‐ups 6 months after hospital discharge so that she remains fine physically. Her clinical manifestations (including dysphagia) have improved within 3 months after discharge, and her bowel movements remain normal.

## Discussion

IMP is an ischaemic colitis characterized by widespread calcification of the mesenteric vein branches and colon wall veins, as well as thickening of the colon wall. Immunological abnormalities, hypercoagulability, and hypertension may be the causes of IMP. Chinese medicines may be involved in the etiology of IMP. A survey in Japan showed that 147 of 222 patients used traditional Chinese medicines.^2^ Previous studies have found that herbal drugs containing gardenia fruit (Sanshishi) are one of the major causes of IMP.^3^ We screened the PubMed database for articles related to “venous sclerosing colitis” or “mesenteric venous sclerosis” and found that IMP can be accompanied by intestinal stenosis,^4^ intestinal necrosis, and intestinal obstruction, and carries a risk of cancerization.^5^ It is easy to misdiagnose or suspect IBD, and the clinical symptoms are atypical and nonspecific. IMP can have a familial incidence, occurring simultaneously in wives and husbands or mothers and children,^6^ which is closely related to the use of Chinese herbal medicines in the family at the same time. Therefore, we support the theory that Chinese medicines are most probably involved in the pathology of IMP. However, if selfheal or any other Chinese medicine is directly involved in the pathogenesis of IMP, this may be determined by further experimentation using a larger dataset.

The diagnosis of IMP relies mainly on typical clinical manifestations, imaging examinations, colonoscopy findings, and histopathological manifestations, which typically include fibrosis and thickening calcification of the vein wall, foam‐like macrophages in the submucosa of small blood vessels, fibroses in the intestinal mucosa, hyaline changes, calcifications, twisting and deformation of mucosal glands, infiltration of lymphocytoplasmic cells, and tissue fibrosis centred on the vein. Esophageal venous sinuses are benign lesions of the esophagus that are mainly generated by the thickening, enlargement, and tortuous twisting of the esophageal wall veins. The formation of esophageal venous sinuses is related to increased pressure in the venous wall. A history of portal hypertension, long‐term chronic inflammation of the esophagus, and increased venous pressure can cause adaptive changes in the venous wall, leading to calcified foci and venous sclerosis.^7^ Calcifications are typically caused by atherosclerosis or ischaemic intestinal changes. In the case where the CT scan indicates multiple visible mesocolon vessel calcifications, calcification is not only limited to the colon but also includes the esophagus. Our patient had multiple calcified shadows in the intestinal wall and multiple calcified foci in the esophageal wall. In addition to congenital factors, portal hypertension, or secondary factors, the formation of esophageal wall calcifications may be related to chronic inflammation, atherosclerosis, and presence of ischaemia in the esophageal wall. Esophageal inflammation, vascular ischaemia, wall calcification, and atherosclerosis may lead to the development of esophageal venous sinuses. Cases of IMP with ischaemic changes in the esophagus and colon are rare.

Long‐term excessive intake of vitamin D (active vitamin D [1,25 hydroxy]) and calcium carbonate can lead to calcium salt deposition, which may increase blood calcium levels and potentially exacerbate arteriosclerosis and vascular calcification, particularly in patients with basic diseases such as hypertension and diabetes. It is worth reminding the patient that he or she should stop calcium intake to avoid exacerbating existing vascular calcification and worsening the progression of IMP disease. Like other hormones, vitamin D has a dual nature, as it is intricately linked to abnormalities in vitamin D metabolism and vascular calcification in various diseases such as atherosclerosis and osteoporosis. Animal experiments have shown that vitamin D toxicity can lead to hypercalcemia and vascular calcification.^8^ Long‐term excessive oral intake of vitamin D may also exacerbate venous calcification processes.^9^ Overuse of vitamin D‐hormone supplements carries significant risks, which has been known for decades. Thus, the traditional clinical manifestations of vitamin D‐hormone toxicity are the same as those of hypercalcemia. As such, studies evaluating the safety of various dosing regimens typically use measurements of serum and urinary calcium to monitor the safety of the administered doses. However, given the number of cell types and tissues that possess 25‐hydroxylase, vitamin D‐hormones may have effects on these systems without necessarily affecting the serum or urinary calcium levels, and all biological processes may be deranged by excess intake. Yet, as discussed above, variations in vitamin D‐hormone production and metabolism may depend significantly on individual genotype, phenotype, and environmental conditions. Thus, a universal upper limit of safety and a universal lower limit of sufficiency for all patients may not necessarily be accurate. Additionally, excess vitamin D‐hormone supplements also displace the active form from binding sites, making it more available even when not appropriate. Further, given the crosstalk with other steroid hormone receptors, vitamin D‐hormones in excess may have physiological effects similar to those of glucocorticoids, estrogen, or even anabolic steroids.^10^ Although their blood calcium and phosphorus levels may be normal, it is an important reminder of the possibility of exacerbating widespread calcification of the venous wall in patients with the IMP diseases against the excessive use of calcium supplements and vitamin D.

In summary, IMP lacks typical clinical manifestations but is easy to diagnose with the support of endoscopy, imaging, and pathological examination. Long‐term use of Chinese medicines (especially selfheal) may promote the initiation and development of IMP. Excessive vitamin D and calcium supplements may lead to the progression of IMP disease. Venous calcification in IMP is not limited to the intestine, as the esophagus may also be affected. However, widespread esophageal vein calcification is difficult to detect and is often overlooked. Reports of esophageal venous sinus are rare and require more attention. Despite the rarity of IMP, it is crucial for physicians to enhance their understanding of the disease and refrain from drug abuse. This proactive approach can effectively minimize the occurrence of IMP and its associated adverse complications.
